# From tradition to evidence: medicinal plants used for childhood diseases in the Ehlanzeni District, Mpumalanga Province, South Africa

**DOI:** 10.3389/fped.2026.1822092

**Published:** 2026-05-11

**Authors:** Celani Lwazi Mkhabela, Boikanyo Calvin Mophuting, Sinorita Chauke, Nobuhle Cynthia Buthelezi, Idowu Jonas Sagbo, Peter Tshepiso Ndhlovu

**Affiliations:** 1 School of Biology and Environmental Sciences, University of Mpumalanga, Mbombela, South Africa; 2 Indigenous Flora Research Group, School of Biology and Environmental Sciences, University of Mpumalanga, Mbombela, South Africa; 3 Cellular Neurobiology, The Salk Institute for Biological Studies, La Jolla, CA, United States

**Keywords:** cytotoxicity, indigenous knowledge, medical ethnobotany, phytochemicals, traditional medicine

## Abstract

Recent studies indicate that about 80% of individuals around the world depend on medicinal plants for healthcare, particularly in rural areas where these plants offer affordability, accessibility, and fewer side effects compared to conventional medicines. Medicinal plants contain phytochemical compounds such as alkaloids, saponins, and tannins, which contribute to their therapeutic properties and are significant to local economies through their commercialization by traditional health practitioners. In Mpumalanga Province, South Africa, many rural communities continue to utilize these plants for various health issues. However, there is limited research regarding the use of medicinal plants for childhood diseases in the region. This study aimed to document the medicinal plants used for treating childhood diseases in the Ehlanzeni District, Mpumalanga Province, South Africa. Data was gathered from online sources, including Google Scholar and Science Direct, using keywords such as cytotoxicity, indigenous knowledge, medical ethnobotany, phytochemicals, and traditional medicine. The inclusion criteria involved studies that examine medicinal plants within the Mpumalanga Province, specifically relating to children aged 0 to 18 years. The study identified 38 plant species from 30 families used for treating 23 childhood diseases. The Fabaceae family was the most common, with roots being the frequently utilized plant part. Decoction was the primary preparation method, and oral administration was the prevalent route. The research found that 12 out of the 38 medicinal plants utilized in the Ehlanzeni District for childhood diseases have not been scientifically examined for their phytochemical properties. Only 31 of the plants have confirmed antibacterial effects, with five showing strong potency (MIC < 15 µg/mL). Moreover, 25 plants were evaluated for their antifungal activity, with *Acokanthera oppositifolia* proving to be the most effective (MIC: 3.9 µg/mL against *Candida albicans*). Furthermore, 25 plants have been previously investigated for their cytotoxic effects, with *Agapanthus africanus* exhibiting significant potency, particularly against SBC-3 cells (IC50 3.7 ± 0.033 μg/mL). The limited scientific validation of phytochemical profiles and biological effects of certain plants highlights the need for further research in this area.

## 1 Introduction

Approximately 80% of the global population rely on medicinal plants for their healthcare needs (1, 2). This reliance highlights the significant and continuing relationship between plants and traditional healing practices. In numerous regions, these plants are not only supplementary treatments; rather, they serve as the primary sources of healthcare, reflecting their great cultural importance and the historical dependence on nature for healing (3). In South Africa, roughly 10% of the country's diverse flora is acknowledged for its medicinal properties, illustrating the wealth of natural resources available for healthcare (4). Many people in the Mpumalanga Province prefer and accept the use of medicinal plants as an alternative to healthcare (5, 6). Medicinal plants are often utilized for their health benefits such as anti-inflammatory, immune-stimulating qualities, and antibacterial properties among others (7).

**Figure 1:**
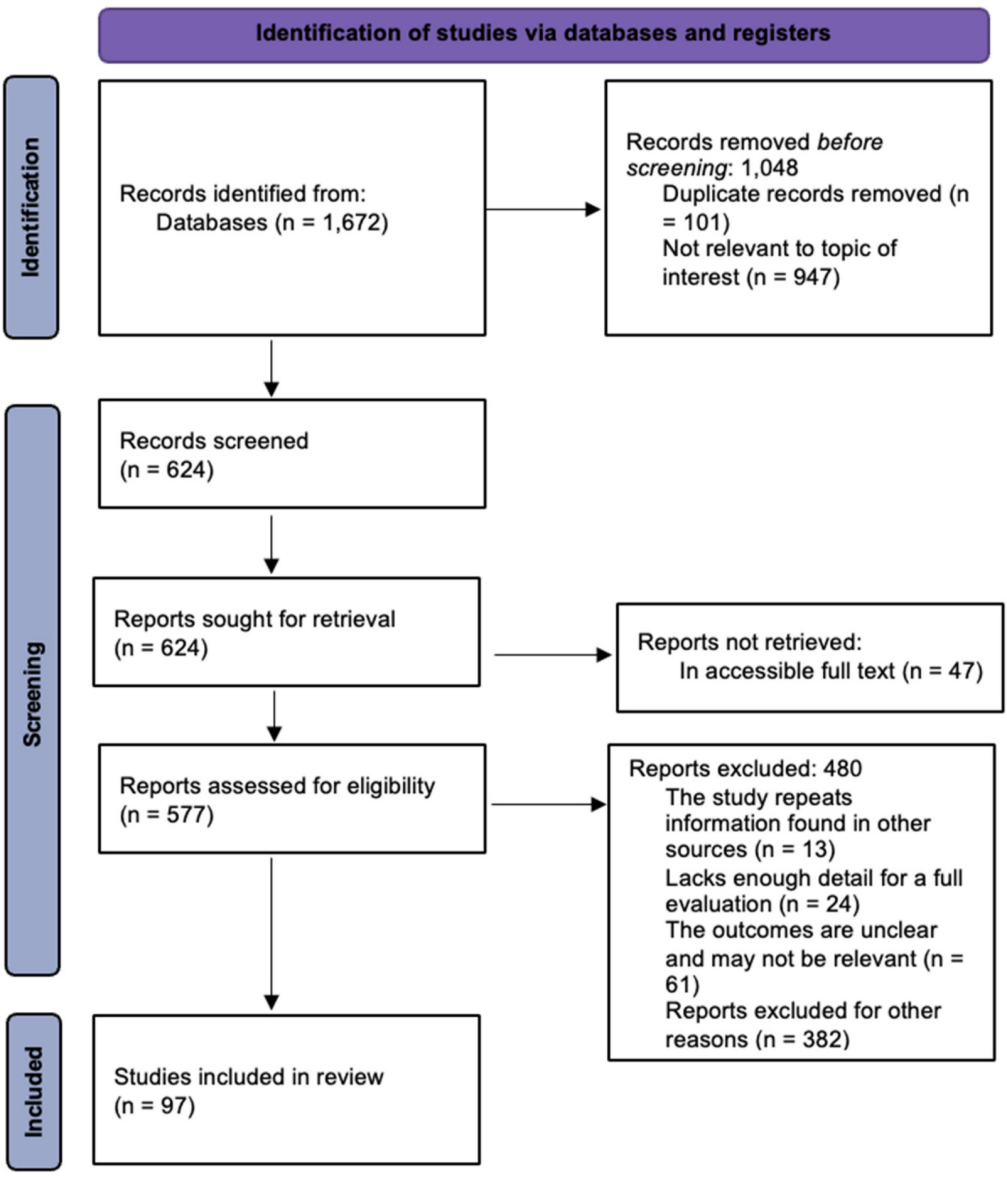
Flow diagram for identification and screening of studies relevant to the current study.

The application of medicinal plants for the management of a wide range of diseases such as neurodegenerative disorder, malaria, hypertension, respiratory infections, gastrointestinal issues, and malnutrition-related conditions among others is based on acceptability, affordability, accessibility and cultural compatibility (8). Traditional medicinal plants are often more accessible to local communities than pharmaceutical medicines (9). Many families can grow these plants in their gardens, or they can easily be harvested from the wild, reducing costs associated with purchasing medicine from pharmacies. There is a growing perception that traditional plant treatments are effective and safer compared to synthetic medications (3, 10). For instance, plants like ginger and peppermint are commonly used for respiratory issues, while leaves of chamomile are employed to treat digestive problems (11). The familiarity with these treatments often leads to a belief in their efficacy. Moreover, Traditional Health Practitioners (THPs) often have a wealth of knowledge about medicinal plants and their uses (12). Parents and caregivers, especially in rural areas, often turn to these THPs for advice and treatment, as they are viewed as trusted sources of health information.

The high plant diversity found in the Mpumalanga Province further contributes to the extensive utilization of medicinal plants across various districts, including the Ehlanzeni District (13, 14). Medicinal plants also offer numerous benefits to people beyond their health advantages. Herbalists, diviners, and street vendors sell these medicinal plants, creating a source of income that helps improve livelihoods. Certain plants may be connected to gods or spiritual beliefs in various societies, which increases their cultural relevance and acceptability (15, 16). In many rural communities, there exists a prevailing mistrust of modern medicines, which can be attributed to past negative experiences (17). This, combined with the high costs and limited accessibility of these modern treatments, often leads individuals to seek alternatives in traditional medicinal plants (9).

South Africa faces significant challenges related to socioeconomic factors, including limited access to healthcare and elevated poverty rates in certain parts of the country (18). In the Mpumalanga Province, particularly the Ehlanzeni District, respiratory infections and poverty related conditions such as malnutrition and diarrhea are prevalent and have a significant impact on children's growth and development (14, 19). Moreover, the high expense of modern medicines contributes to the overall difficulties in effectively addressing paediatric and public health needs within the region (20). In addition, numerous synthetic medications have been documented as having the potential to cause undesirable side effects. For example, antibiotics such as amoxicillin are widely prescribed for treating bacterial infections such ear infections, pneumonia, and strep throat, but they can lead to gastrointestinal disturbances, including diarrhea and nausea, and may also provoke allergic reactions in some children (21, 22). Moreover, Inhaled corticosteroids (ICS) are often considered a first-line treatment option for children with frequent symptoms or moderate to severe asthma. However, these ICS can sometimes result in oral thrush or respiratory infections (23). Similarly, cough syrups, often containing dextromethorphan or guaifenesin, may lead to side effects like drowsiness, dizziness, or stomach upset (24). Over-the-counter cough and fever medications often contain active ingredients such as antihistamines, decongestants, antitussives, expectorants, and analgesics-antipyretics (25). The combination of multiple ingredients in these products increases the risk of adverse effects and unintentional overdoses.

While traditional medicine is widely practiced, there is often insufficient formal documentation of local knowledge regarding specific medicinal plants used for treating childhood diseases (26). This can lead to valuable information being lost or underutilized. Many medicinal plants used in the Ehlanzeni District have not undergone rigorous scientific studies to validate their efficacy and safety in treating childhood diseases (20). This lack of clinical research makes it challenging for health practitioners to recommend these treatments confidently. In Ehlanzeni District, most existing research on medicinal plants tends to focus on adult populations or general health benefits, with little emphasis placed on their specific applications and effects in children (6, 14, 27). This gap limits the development of herbal treatments for pediatric care. There is a lack of comprehensive studies to assess the safety and potential toxicity of medicinal plants used by caregivers for children. Thus, the aim of this study is to gather and document information about the medicinal plants used to treat and manage childhood diseases in the Ehlanzeni District, Mpumalanga Province.

## 2 Methodology

This study was conducted from January 2024 to December 2025 to collect information on medicinal plants used to treat and manage childhood diseases in the Ehlanzeni District of Mpumalanga Province, South Africa. The search strategy for this systematic review involved a comprehensive and multi-step approach. Initial steps included defining keywords such as cytotoxicity, indigenous knowledge, medical ethnobotany, phytochemicals, and traditional medicine. A combination of search terms was developed using Boolean operators (AND, OR, NOT) to ensure a thorough search across databases, including Science Direct, PubMed, and Google Scholar. Grey literature was also explored through institutional repositories, conference proceedings, and relevant governmental and non-governmental organization publications to gather additional insights.

An inclusion criterion was established to ensure that only pertinent articles were consulted for information gathering. Consequently, articles were selected based on their specific focus on medicinal plants utilized in Mpumalanga Province, particularly within the Ehlanzeni District. Research studies targeting paediatric populations, specifically children aged 0–18 years, and examining the use of medicinal plants for the treatment of various diseases were included in this review. Preference was given to peer-reviewed journal articles, theses, and dissertations to uphold the credibility and academic rigour of the selected sources. The exclusion criteria eliminated studies irrelevant to the defined geographical area and those not focused on childhood diseases. The primary language of interest was English, and all selected publications were required to provide an abstract in English. Any studies in languages other than English were excluded from the review to maintain coherence. The data extraction process involved a structured and meticulous approach, conducted independently by four researchers to minimize bias and enhance reliability. Each utilized a standardized data extraction form to collect key information, including study characteristics (authors, year of publication, study design), details about the medicinal plants (species names, preparation methods, administration techniques, phytochemical compounds, and antifungal, antibacterial, and cytotoxicity properties of the plants), and specific childhood diseases being addressed. Disagreements between the researchers were resolved through discussion and consensus, and a fifth researcher was consulted whenever necessary.

The quality of the studies included in this systematic review was assessed using a risk-of-bias assessment framework, systematically examining study objectives, methodology appropriateness, recruitment strategies, and data collection processes. Each study was rated for potential biases, including selection and reporting bias, based on established criteria to assign ratings for comparative analysis across studies. Studies demonstrating strong methodological practices received higher quality ratings, while those with substantial limitations were flagged for potential bias. This quality assessment was done to enhance the credibility of the review findings and identify gaps in existing literature. The comprehensive search of online databases yielded a total of 1,672 articles to gather data for the study. Following the initial review, 624 articles were screened after the removal of duplicates and those deemed irrelevant to the research focus. Subsequent exclusion of articles with ambiguous outcomes, those without accessible full texts, and those lacking sufficient information for thorough evaluation resulted in a final selection of 97 articles. These selected articles were used in the study to identify medicinal plants employed for the management of childhood diseases in the Ehlanzeni District of Mpumalanga Province, South Africa (Table 1). The scientific names of the plants were verified using “WFO (2025): World Flora Online. Published on the Internet; http://www.worldfloraonline.org. Accessed on: January 2024 to December 2025”.

**Table 1:** Inventory of medicinal plants used for childhood diseases in the Ehlanzeni District, Mpumalanga Province, South Africa.

S/N	Scientific & family name	Life form	Plant part used & condition(s) treated	Preparation method	Mode of administration	References
1.	*Abrus precatorius* subsp. Africanus Verdc. Fabaceae	Climber	Roots are used to treat conditions associated with nail-biting, such as onychophagia and koilonychia	Decoction	Orally	(14)
2.	*Vachellia karroo* (Hayne) Banfi & Galasso Fabaceae	Tree	Roots are used to treat conditions associated with nail-biting, such as onychophagia and koilonychia	Decoction	Orally	(14)
3.	*Acokanthera oppositifolia* (Lam.) Codd Apocynaceae	Tree	Leaves are used to treat mental illness conditions such as memory loss, headache, epilepsy	Powdered Infusion	Snuff Orally	(28)
4.	*Acorus calamus* L. Acoraceae	Herb	Roots are used to treat fever, loss of appetite, intestinal worms	Decoction	Orally	(28)
5.	*Adenia gummifera* (Harv.) Harms var. gummifera Passifloraceae	Climber	Bulbs are used to treat colic	Decoction	Orally	(14)
6.	*Agapanthus africanus* (L.) Hoffmanns. subsp. Africanus Agapanthaceae	Shrub	Roots are used to treat resuscitation, nail-biting	Decoction	Orally Topical—bath inhale smoke	(14)
7.	*Alepidea amatymbica* Eckl. & Zeyh. Apiaceae	Herb	Roots are used to treat snake bites	Powdered	Inhale smoke Snuff Topically	(28)
8.	*Allium sativum* L. Alliaceae	Herb	Leaves are used to treat colic	Decoction	Orally	(14)
9.	*Aloe ferox* Mill. Asphodelaceae	Succulent	Leaves are used to treat skin irritations, toothache	Infusion	Orally	(28)
10.	*Annona senegalensis* Pers. subsp. Senegalensis Annonaceae	Shrub	Barks are used to treat sunken fontanelle, mental illness conditions such as memory loss. Roots are used to treat toothache	Decoction	Orally	(14)
11.	*Ansellia africana* Lindl. Orchidaceae	Epiphytes	Leaves are used to treat sunken fontanelle	Decoction	Orally	(14)
12.	*Artemisia afra* Jacq. ex Willd. Asteraceae	Shrub	Leaves are used to treat fever	Decoction	Orally Steaming	(28)
13.	*Asparagus aethiopicus* L Asparagaceae	Climber	Roots are used to treat body pains	Decoction	Orally	(28)
14.	*Bulbine frutescens* (L.) Willd. Asphodelaceae	Shrub	Bulbs are used to treat sunken fontanelles, typhoid	Decoction	Orally	(14)
15.	*Carica papaya* L. Caricaceae	Herb	Roots are used to treat haematuria and to revive children from unconsciousness	Decoction	Orally Topical—bath inhale smoke	(14)
16.	*Clematis brachiata* Thunb. Ranunculaceae	Climber	Roots are used to treat sunken fontanelles	Smoke	Orally	(14)
17.	*Coix lacryma-jobi* L. Poaceae	Herb	Seeds are used to ease pain associated with teething	Worn	Worn (necklace or waist beads)	(20)
18.	*Cussonia transvaalensis* Reyneke Araliaceae	Tree	Roots are used for improving weight	Decoction	Topical	(14)
19.	*Diospyros mespiliformis* Hochst. *ex* A.DC. Ebenaceae	Tree	Roots are used for sunken fontanelle	Decoction	Orally	(14)
20.	*Euclea crispa* (Thunb.) Gurke subsp. *Crispa* Ebenaceae	Tree	Roots are used to treat sunken fontanelle	Decoction	Orally	(14)
21.	*Fallopia convolvulus* (L.) Á.Löve Polygonaceae	Herb	Roots are used to treat colic	Decoction	Orally	(14)
22.	*Gomphocarpus fruticosus* (L.) Aiton f. subsp. *Fruticosus* Apocynaceae	Shrub	Roots are used to treat sunken fontanelles	Decoction	Orally	(14)
23.	*Gymnosporia senegalensis* (Lam.) Loes. Celastraceae	Shrub	Roots are used to treat sunken fontanelles	Decoction	Orally	(14)
24.	*Hibiscus pusillus* Thunb. Malvaceae	Herb	Barks are used to treat sunken fontanelles	Decoction	Orally	(20)
25.	*Hypoxis hemerocallidea* Fisch., C.A.Mey. & Ave-Lall Hypoxidaceae	Herb	Roots are used to treat sunken fontanelles	Decoction	Orally	(14)
26.	*Lannea schweinfurthii var. stuhlmannii* Engl. Anacardiaceae	Shrub	Leaves are used to treat boils	Decoction	Topically	(28)
27.	*Lantana rugosa* Thunb. Verbenaceae	Shrub	Roots are used to treat sunken fontanelles	Decoction	Orally	(14)
28.	*Leonotis ocymifolia* (Burm.f.) Iwarsson Lamiaceae	Shrub	Roots are used to treat sunken fontanelles	Decoction	Orally	(14)
29.	*Lippia javanica* (Burm.f.) Spreng. Verbenaceae	Shrub	Roots and leaves are used to treat sunken fontanelles	Decoction	Orally	(14)
30.	*Lycopersicon esculentum* L. Solanaceae	Herb	Leaves are used to treat typhoid	Decoction	Orally Enema	(14)
31.	*Prunus persica* (L.) Batsch Roseaceae	Tree	Leaves are used to treat typhoid	Decoction	Orally Enema	(14)
32.	*Scabiosa columbaria* L. Dipsacaceae	Herb	Roots are used to treat colic	Decoction	Orally	(20)
33.	*Schizocarphus nervosus* (Burch.) Van der Merwe Hyacinthaceae	Herb	Bulbs are used to treat a reddish spot at the back of the neck of babies	Decoction	Orally	(20)
34.	*Sclerocarya birrea* subsp. Caffra Anacardiaceae	Tree	Barks are used to treat diarrhea. Fruit/seeds are used to treat hiccups	Decoction	Orally, topically, smoking, worn	(14)
35.	*Senna occidentalis* (L.) Link Fabaceae	Shrub	Roots are used to treat colic, sunken fontanelles	Decoction	Orally	(14)
36.	*Sida acuta* Burm.f. subsp. Acuta Malvaceae	Herb	Roots are used to treat sunken fontanelles	Decoction	Orally	(14)
37.	*Siphonochilus aethiopicus* (Schweinf.) B.L.Burtt Zingiberaceae	Herb	The whole plant is used to treat typhoid	Decoction	Orally Topically—bath inhale smoke	(14)
38.	*Warburgia salutaris* (Bertol.f.) Chiov Canellaceae	Tree	The barks are used to treat tonsils, influenza, fever	Infusion	Orally	(28)

## 3 Results and discussion

### 3.1 Inventory of medicinal plants used for childhood diseases in the Ehlanzeni District

The present study discovered that local communities in the Ehlanzeni District widely use medicinal plants for the treatment of childhood diseases, with 38 distinct species identified for this purpose. Table 1 presents comprehensive information detailing the specific plants and their respective parts utilized, the diseases they are associated with, and the methods of preparation and administration employed in their application. As far as we could ascertain, only three studies have explored the use of medicinal plants for the treatment of childhood diseases in two local municipalities within the Ehlanzeni District, namely Bushbuckridge and Nkomazi. These studies have contributed valuable insights into the traditional practices and herbal remedies employed by local communities in addressing various childhood diseases. However, it is important to note that the other two municipalities within the Ehlanzeni District, Mbombela and Thaba Chweu, have yet to be investigated regarding their medicinal plant usage for treating childhood diseases.

### 3.2 Taxonomic distribution of medicinal plant families

**Figure 2:**
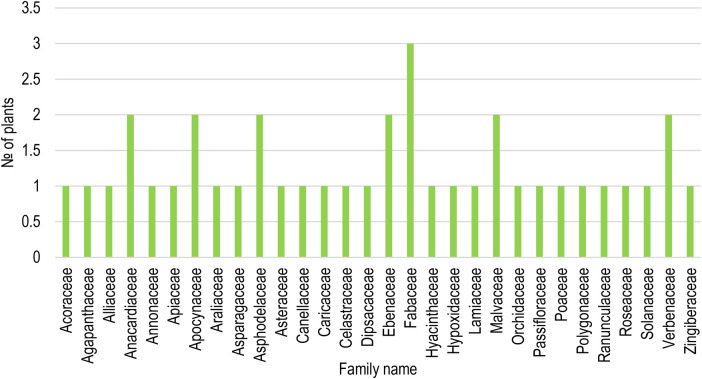
Number of medicinal plants used from each plant family in the Ehlanzeni district, Mpumalanga Province, South Africa.

These species are classified into 30 distinct families (Figure 2). Notably, the Fabaceae family was the most common among the plant families, represented by three species of medicinal plants: *Abrus precatoriu*s, *Vachellia karroo*, and *Senna occidentalis*. The second most common were Anacardiaceae, Apocynaceae, Asphodelaceae, Ebenaceae, Malvaceae, and Verbenaceae families, each contributing two species. This discovery conforms with the findings of Khumalo et al. (5); Mashile et al. (14); Tahir et al. (29), who similarly reported the Fabaceae family as the most commonly utilized plant group for medicinal purposes. The Fabaceae family consists of a vast range of species, providing a multitude of options for medicinal uses. This diversity allows for adaptability to various climates and cultural practices. Laftouhi et al. (30) cited that the prominence of the Fabaceae family in traditional medicine may be attributed to their resilience in diverse environmental conditions, nutrient-rich characteristics, and effectiveness in addressing a broad range of health issues. Saikia et al. (31) also explained the dominance of this family by stating that the Fabaceae, also known as the legume family, is a big family that includes a diverse range of herbs, shrubs, climbers, and trees, comprising more than 19,000 species. This makes it the third-largest group of flowering plants, following Orchidaceae and Asteraceae (32).

### 3.3 Plant parts utilized in the treatment of childhood diseases

**Figure 3:**
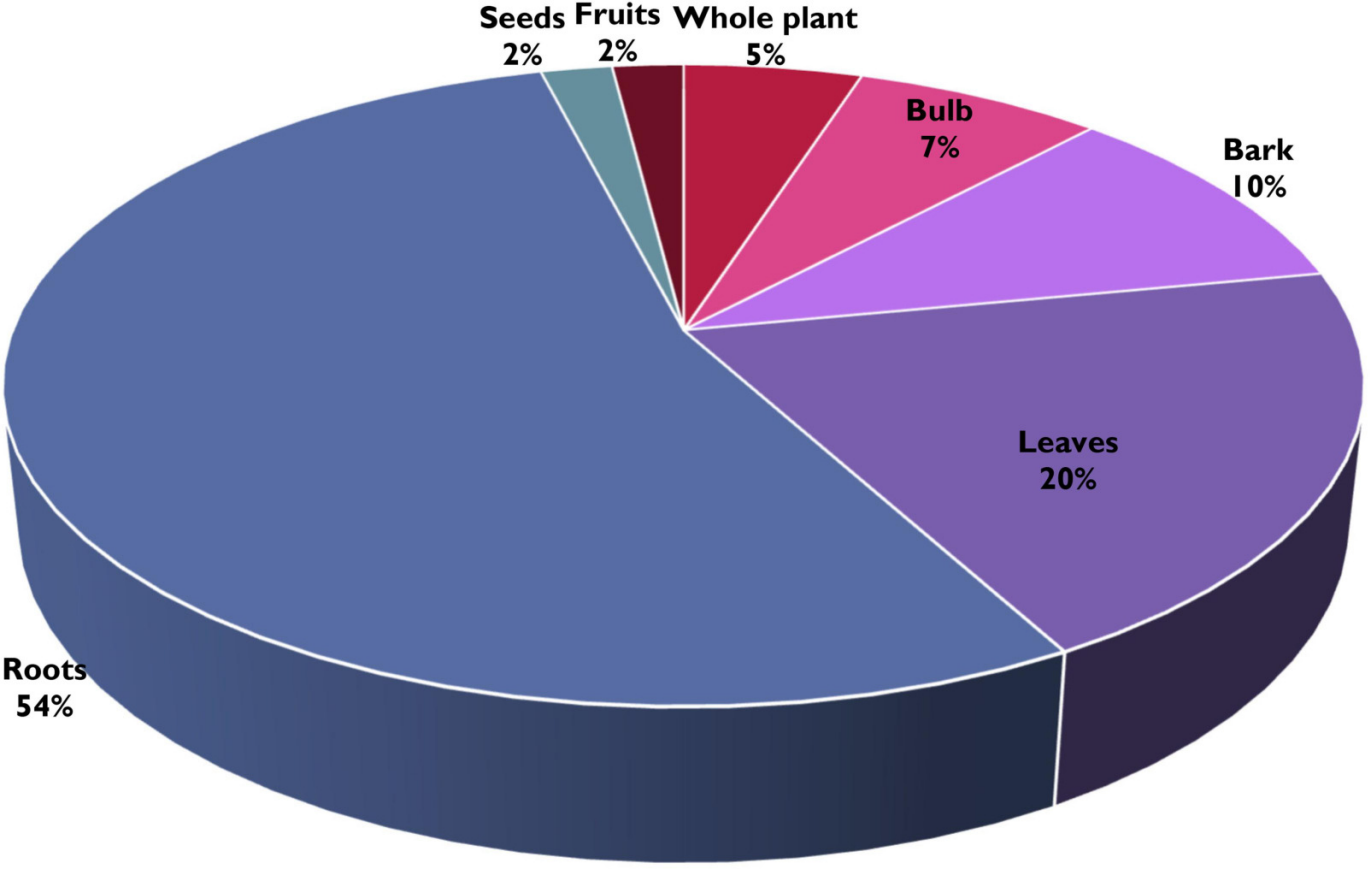
Plant parts that are used to treat childhood diseases in the Ehlanzeni District, Mpumalanga Province, South Africa.

In the Ehlanzeni District, the roots are the most frequently utilized part of medicinal plants, representing 54% of their usage (Figure 3). The leaves follow this at 20%, the bark at 10%, the bulbs at 7%, the whole plant at 5%, and seeds and fruit contributing 2% each. Maroyi (26) equally recorded that roots are the main plant part used in formulating treatments for childhood diseases in Zimbabwe. Correspondingly, Ndhlovu et al. (33) reported that in the North West Province of South Africa, treatments for childhood diseases predominantly incorporate roots and rhizomes (40%), followed by leaves (23%) and whole plants (20%). Khoza (28) explained that roots typically store a high concentration of phytochemical compounds, such as alkaloids, glycosides, and essential oils, responsible for the plant's therapeutic effects hence they are frequently utilized in traditional medicine. Maroyi (26) also clarified that these compounds can be more concentrated in roots compared to other parts of the plant.

Roots function as storage organs for nutrients and phytochemical compounds and are accessible throughout the year, particularly those of perennial plants (34). This availability contrasts with other plant parts, such as fruits, flowers, and leaves, typically present only during specific seasons, further encouraging the use of roots. Moreover, by being underground, roots are protected from sunlight, wind, and other environmental factors which can diminish the fragile medicinal compounds present in parts of the plant exposed to the environment such as leaves or flowers (35). This protection helps ensure the stability and durability of the plant's medicinal qualities, making them a more dependable source for traditional medical treatments. However, the extensive reliance on root harvesting may pose a significant threat to local biodiversity and necessitate conservation efforts. The collection of roots from medicinal plants, particularly small herbs, typically entails the uprooting of entire plants. This practice not only diminishes their populations but also disrupts the local ecosystems that depend on these plants for their stability and diversity.

### 3.4 Growth forms and life forms of the identified medicinal plants

**Figure 4:**
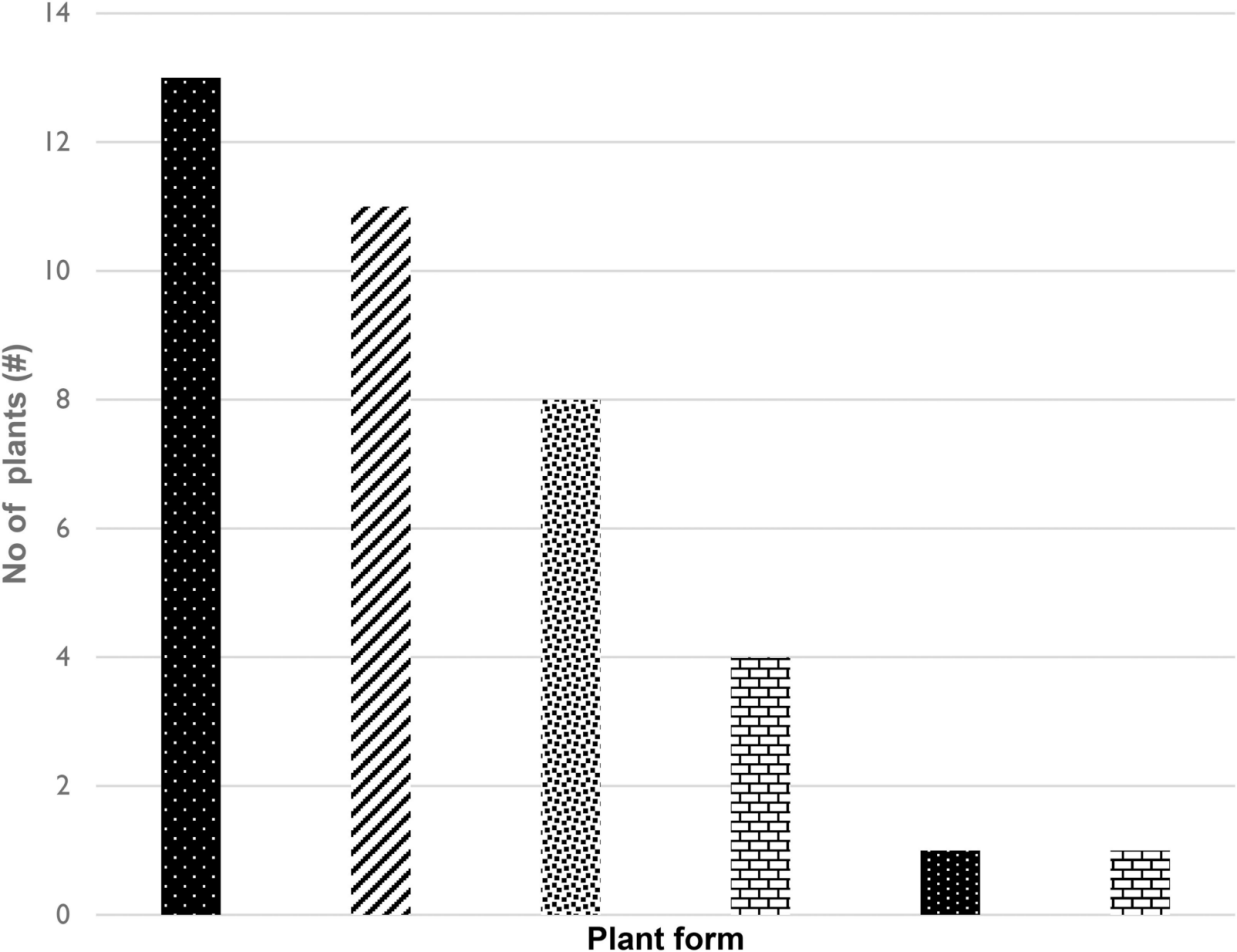
Frequency of life forms of plants utilized in the Ehlanzeni District. Mpumalanga Province, South Africa.

The study found that herbs are the predominant life form utilized in the Ehlanzeni District for the treatment of childhood diseases, comprising thirteen distinct species (Figure 4). Following closely are shrubs represented by eleven species and trees represented by eight species. In addition, climbers contribute four species, while epiphytes and succulents are represented by just one species each. This distribution of plant types aligns with observations made by Khoza (28), who highlighted that trees, shrubs, and herbs are the most frequently employed by traditional healers. In the same way, Maroyi (26) reported that herbs (32%), shrubs (30%), and trees (24%) are the primary sources of medicinal plants used to treat and manage childhood diseases in Zimbabwe. The dominance of these plant life forms may reflect the types of vegetation found in the Ehlanzeni District, an area known for its diverse plant life, which includes forests, woodlands, grasslands, and savannahs, along with a significant presence of thick bushland.

### 3.5 Methods of preparation for pediatric herbal treatments

**Figure 5:**
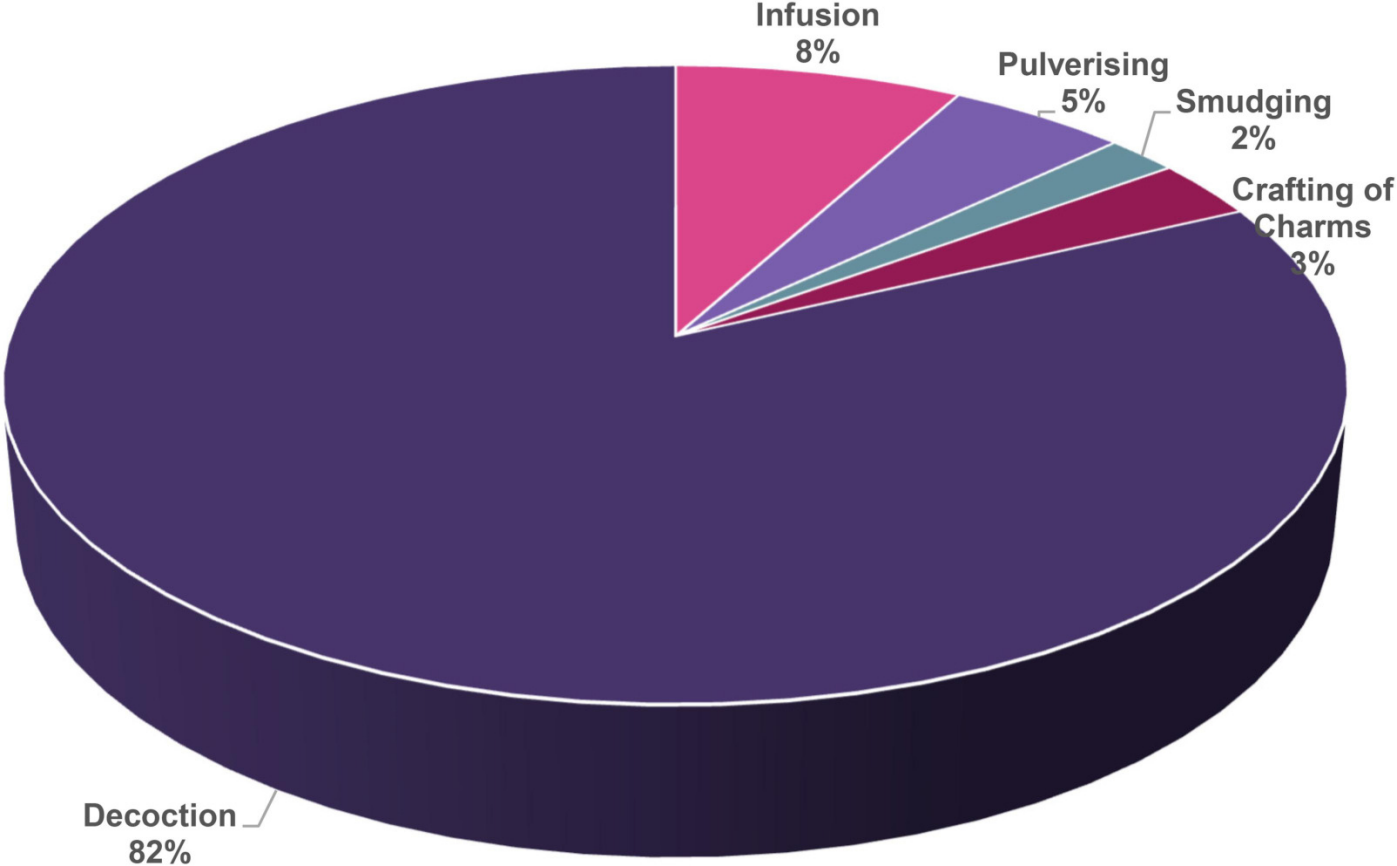
Methods of preparing treatments for treating childhood diseases in Ehlanzeni District, Mpumalanga Province, South Africa.

The current study revealed that five different methods are used by the people in the Ehlanzeni District, Mpumalanga Province, South Africa to prepare treatments for treating childhood diseases using medicinal plants (Figure 5). Among these methods, decoction, a process of extracting valuable compounds by boiling plant materials in water, was the commonly used preparation method (82%). This method (boiling) helps eliminate potential microbes and bacteria present in water or plant material, thereby enhancing safety, particularly for children (36). Other preparation methods include infusion (8%), pulverizing—reducing plant material into a fine powder (5%), crafting of charms/amulets (3%), and smudging—burning plant material to produce smoke (2%). These findings are in accordance with previously published studies, which have also cited decoctions, powdered plant preparations and infusions as the most frequently used method of preparations in Southern Africa (5, 37, 38). Parents and caregivers choose and select the most suitable method based on the specific plant part used and the medical or spiritual issues being addressed, and the desired effect. In the current study, the dominance of the decoction method aligns with the trend of the people in the study area to rely more on roots. Evbuomwan et al. (39) mentioned that the decoction preparation method is favoured for its simplicity and is commonly used as it is believed that boiling medicinal plants, typically more challenging parts of plants, like roots and bark, is the most efficient way to extract their phytochemical compounds.

### 3.6 Modes of administration for childhood treatments

**Figure 6:**
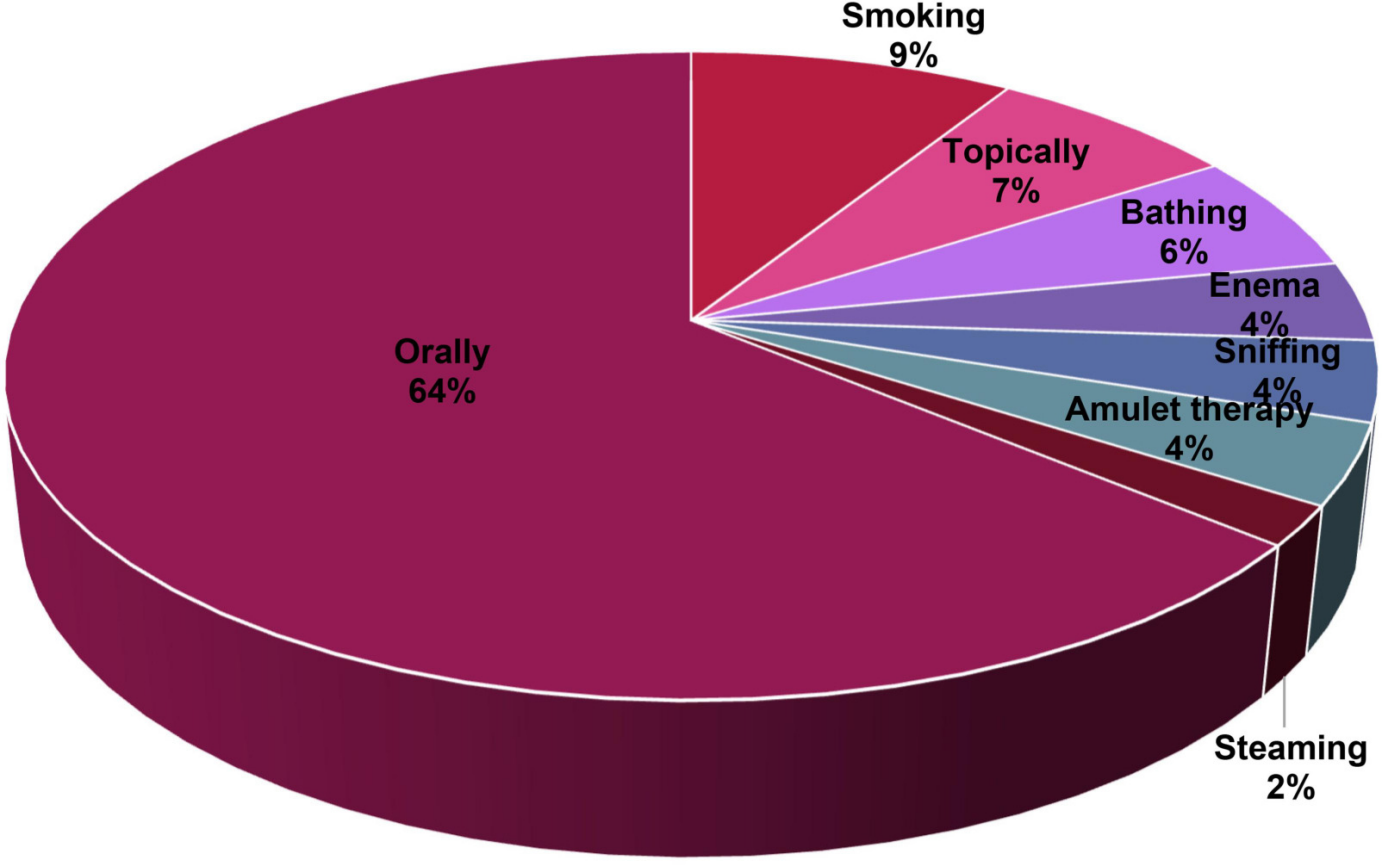
Methods of administering treatments to children in Ehlanzeni District, Mpumalanga Province, South Africa.

Oral administration was the predominant method for delivering medication to children in the Ehlanzeni District, accounting for 64% of cases observed (Figure 6). Smoking is the second most common method of treatment administration, accounting for 9% of usage. Topical application ranks third, with 7% of the plants being prepared and applied directly to the skin, particularly for skin irritations and inflammation. Additional methods of remedy administration include bathing (6%), enemas (4%), sniffing (4%), and wearing the plant or plant part around the neck or waist (4%), as well as steaming (2%). The finding of the current study supports the discoveries of Khoza et al. (20), who correspondingly discovered that oral administration was the most common route of administration for the treatment of maternal and childhood health related diseases. Recent studies by Che et al. (40); Eshete and Molla (41); Kachmar et al. (42) have reported that oral administration of medication is generally the most straightforward and most familiar method of taking medicine. Similarly, Lou et al. (43) explained that this mode of administration allows for easy self-administration without needing professional help or special equipment.

### 3.7 Categories of childhood diseases treated in the Ehlanzeni District, Mpumalanga Province, South Africa

**Figure 7:**
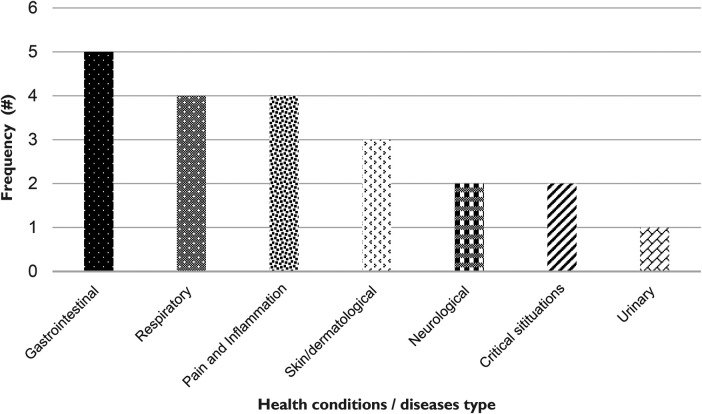
Frequency of childhood diseases for different categories of diseases in the Ehlanzeni District, Mpumalanga Province, South Africa.

The study identified 23 childhood diseases that are commonly encountered and treated with medicinal plants in the Ehlanzeni District, Mpumalanga Province, South Africa. These diseases are grouped into nine categories (Figure 7). Notably, most of these childhood diseases (accounting for 21% of the total) fall within the gastrointestinal category, which involves five specific diseases. This finding aligns with a study conducted in Algeria, which revealed a prevalence of gastrointestinal conditions that are managed effectively due to the extensive variety of medicinal plants utilized in their treatment (44). The gastrointestinal conditions affecting children in the Ehlanzeni District include diarrhea, which is one of the leading causes of morbidity and mortality in young children globally, especially in rural communities where access to clean water and sanitation is limited (45). According to the World Health Organization (46), diarrhea is the third leading cause of death in children under 5 years old and is responsible for killing around 443,832 children every year.

The other gastrointestinal conditions are colic, which is characterized by severe abdominal pain; typhoid, a serious bacterial infection; loss of appetite, which may signal underlying health issues; and intestinal worms, a common parasitic infection among children. Previous studies have highlighted the prevalence and high occurrence of these childhood conditions, particularly in rural areas (47–49). Six plant species were identified as treatments for these gastrointestinal issues in the study area: *Adenia gummifera*, *Allium sativum*, *Fallopia convolvulus*, *Scabiosa columbaria*, *Sclerocarya birrea*, and *Senna occidentalis*.

Respiratory diseases, along with pain and inflammation conditions, rank as the second most prevalent categories of diseases, each contributing four distinct health issues to the overall count. In the current study, the respiratory conditions treated include fever, influenza, persistent hiccups, and tonsillitis. Osadolor et al. (50); Reilly (51) reported that a high prevalence of these conditions is observed in children from rural communities due to factors like lower vaccination rates, limited access to healthcare, higher exposure to infectious agents, and challenges in maintaining hygiene. In the Ehlanzeni District, four notable plant species have been documented for their therapeutic properties in addressing these conditions: *Acorus calamus*, *Artemisia afra*, *Sclerocarya birrea*, and *Warburgia salutaris*. The use of these plants for the treatment of the reported respiratory infections is widely documented (52–55).

The pain and inflammatory category include various childhood conditions such as body pains, headaches, toothaches, and teething discomfort. Nguyen and Caine (56) explained that body pains in children can arise from varied causes, including growth spurts, injuries, or infections. In rural settings, children may be more active outdoors, increasing the likelihood of minor injuries or strains. The entry of headache affecting children across the study area is supported by Ademi and Ademi (52); Khan et al. (57), who stated that headaches are common in children and can be triggered by several factors, including dehydration, tension, stress, or even environmental elements such as noise and light. Moreover, access to dental care is often limited in rural regions, and parents may lack knowledge regarding dental hygiene (58). This can lead to a higher prevalence of tooth decay and associated pain among children. Teething is a normal developmental process that typically occurs in infants and toddlers, causing discomfort as new teeth push through the gums (59). To address these issues, five medicinal plants have been utilized by the communities within Ehlanzeni District: *Acokanthera oppositifolia*, *Aloe ferox*, *Annona senegalensis*, *Asparagus aethiopicus*, and *Coix lacryma*-jobi.

The dermatological category was linked to three childhood diseases in the Ehlanzeni District. The skin issues reported included boils, which are typically caused by bacterial infections affecting hair follicles and can be painful; skin irritations, which may arise from allergies or irritants; and a red spot on the back of an infant's neck, which could indicate several problems, such as heat rash or an allergic reaction (60–62). A recent study reported that skin-related conditions account for approximately 34% of all diseases observed in rural populations (63). Globally, skin diseases represent the fourth leading cause of non-lethal disease burden, often leading to significant disability and a diminished quality of life (64). The local medicinal plants used to treat skin conditions in the Ehlanzeni District are *Lannea schweinfurthii*, *Aloe ferox*, and *Schizocarphus nervosus*. Among these plants, *A. ferox* is known for its effectiveness in treating various skin conditions. Its gel is known for its soothing, anti-inflammatory, and healing properties, making it beneficial for treating issues like burns, cuts, and skin irritations (65).

Three childhood conditions (sunken fontanelle, nail-biting, and poor growth or weight gain) are categorized under developmental and behavioral issues. These conditions can affect a child's ability to manage or control emotions and behaviors. For instance, nail-biting is a common behavior often linked to anxiety or stress (66). A sunken fontanelle may indicate dehydration or nutritional problems, while poor growth could signal feeding issues or other health concerns (67). In the Ehlanzeni District, nineteen medicinal plants are used to address these conditions; notably, fifteen of these plants are specifically employed to treat sunken fontanelle. According to Kannikeswaran (68) and Lipsett et al. (67), while sunken fontanelle itself does not directly lead to death, it is a concerning indicator of an underlying issue, most commonly dehydration or malnutrition, which significantly contribute to the mortality and morbidity of children under five years old in low-middle-income countries.

Resuscitation and snake bites are categorized as critical situations because they are life-threatening emergencies that require immediate action and intervention. Resuscitation can occur for various reasons, such as birth asphyxia, cardiac arrest, or severe respiratory distress (69). Resuscitation refers to a set of emergency medical procedures performed to restore normal breathing and circulation in infants experiencing respiratory distress. Recent study confirms that steam inhalation using specific medicinal plants may assist in alleviating respiratory difficulties by helping to clear congestion and enhance airflow (70). In the Ehlanzeni District, two medicinal plants commonly used for resuscitation are *Agapanthus africanus* and *Carica papaya*. Rural areas often have greater exposure to wildlife, including snakes, which increases the risk for children playing or exploring in fields, gardens, or near bodies of water. Symptoms of snake bites can vary depending on the species of snake but may include pain, swelling, discoloration, difficulty breathing, and in severe cases, shock or even death (71). In Ehlanzeni District, people use *Alepidea amatymbica* to manage snake bites. Maroyi et al. (72) confirmed the application of *Alepidea amatymbica* as traditional treatment for snake bites in Southern Africa.

The neurological diseases category was represented by two sicknesses, epilepsy and mental illness. Notably, *Acokanthera oppositifolia* is employed by the locals in the study area for the treatment of epilepsy, a neurological disorder characterized by recurrent seizures that can vary in frequency and severity (73). Additionally, *Annona senegalensis* is used to support mental health in children, particularly in addressing anxiety diseases and depression, which may arise from various factors, including the loss of a family member. The least represented disease category was urinary issues, with only one condition reported (haematuria). Haematuria refers to the presence of blood in the urine and may indicate several underlying conditions, including urinary tract infections and kidney diseases (74). For this condition, *Carica papaya* is utilized as a treatment option. Correspondingly, Barnabas et al. (75); Yeung (49) reported that the leaf extract of *Carica* papaya is recognized for urinary support, helping alleviate symptoms such as frequent urination, haematuria, burning sensations, and inflammation in the urinary tract.

### 3.8 Phytochemical constituents

The current study reveals that out of 38 medicinal plants utilized in the Ehlanzeni District for the treatment of childhood diseases, 26 have undergone scientific investigation to identify the presence of phytochemical compounds. Conversely, 12 plants have not been thoroughly validated through rigorous scientific methods or have limited comprehensive documentation in the existing literature. A detailed list of the plants that have been scientifically validated for their phytochemical compounds is provided in Table 2.

**Table 2:** Phytochemical compounds of the medicinal plants used in the Ehlanzeni District.

S/N	Scientific name of plant	Phytochemical constituents
1.	*Acokanthera oppositifolia*	Palmitate, lupeol, acovenoside A, β-sitosterol (76)
2.	*Acorus calamus*	principle A- and β-asarone (77–79)
3.	*Agapanthus africanus*	Saponin, (80)
4.	*Alepidea amatymbica*	phenol, flavonol, flavonoid, tannin, proanthocyanidin, saponin, and alkaloid (81)
5.	*Allium sativum*	alkaloid, saponins, flavonoids, glycoside, anthraquinones, tannin and terpenoids (82)
6.	*Aloe ferox*	aloeresin A, aloesin and aloin (83, 84)
7.	*Ansellia africana*	linoleic acid, linolenic acid, *p*-cresol, eicosane, n-butyl acetate, heptadecane, 2-pentanone, and styrene (85)
8.	*Artemisia afra*	camphor, 1,8-cineole, artemisia ketone, artemisyl acetate, and camphene (86)
9.	*Asparagus aethiopicus*	alkaloids, flavonoids, glycosides, saponins, sterols, tannins (87)
10.	*Bulbine frutescens*	flavonoids and terpenoids (88)
11.	*Carica papaya*	Oleic acid, Octadecanoic acid, (47)
12.	*Clematis brachiata*	Tanins, (89)
13.	*Coix lacryma-jobi*	Dodecanoic acid (90)
14.	*Cussonia transvaalensis*	Triterpenoids (91)
15.	*Euclea crispa*	catechin, epicatechin, gallocatechin, hyperoside, quercitrin, (92)
16.	*Fallopia convolvulus*	Sterols, phenols, anthraquinones, chromone, stilbenes, amides, flavonoids (93, 94)
17.	*Hypoxis hemerocallidea*	Phenolics, flavonoids, (95)
18.	*Lantana rugosa*	alkaloid lantanin, β-caryophyllene, caryophyllene oxide, neral and geranial, (96)
19.	*Leonotis ocymifolia*	methyl linolenate, n-hexadecanoic acid, phytol, octadecenoic acid methyl ester, (97)
20.	*Prunus persica*	phenolics, flavonoids, anthocyanins, and procyanidins (98)
21.	*Scabiosa columbaria*	alkaloids, flavonoids, phenolics, tannins, steroids, terpenoids, and saponins (99)
22.	*Senna occidentalis*	cyperene, β-caryophyllene, limonene and caryophyllene oxide (100)
23.	*Sida acuta*	free flavonoids, ethyl acetate (bound flavonoids, (101)
24.	*Siphonochilus aethiopicus*	Siphonochilone, 1,8-cineole, cis-allo-ocimene and (E)-β-ocimene, (102)
25.	*Vachellia karroo*	(saponins and cardiac glycosides)—(103)
		(Epicatechin 1, β-sitosterol 2 and epigallocatechin)—(104)
26.	*Warburgia salutaris*	Salutarisolide, (105)

Phytochemical compounds in medicinal plants play a vital role in traditional medicine by providing therapeutic benefits that have been known for centuries (106). These compounds often exhibit various biological activities, including anti-inflammatory, antimicrobial, and antioxidant properties, which are essential for treating a wide range of diseases. For instance, *Hypoxis hemerocallidea and Lantana rugosa* are rich in alkaloids, glycosides, flavonoids, and ribosome-inactivating proteins (RIPs) (95, 96). These compounds contribute significantly to the plants’ antimicrobial and anticancer potential, as highlighted by various research studies (107, 108). El-Saadony et al. (109) mentioned that antimicrobial compounds found in various plants can help combat infections such as respiratory diseases, gastroenteritis, and skin infections, providing essential treatment for children who may be more susceptible to these conditions. Additionally, anticancer properties may aid in the management of pediatric cancers, such as leukemia and brain tumors, by inhibiting the growth of cancer cells and reducing side effects associated with conventional therapies (110, 111).

*Cussonia transvaalensis* and *Bulbine frutescens* were found to possess triterpenoids (88, 91). These compounds are known for their anti-inflammatory and hepatoprotective effects (112, 113). Conditions such as asthma, juvenile arthritis, and inflammatory bowel disease can be alleviated due to the anti-inflammatory effects of triterpenoids of these plants, helping to reduce swelling, pain, and discomfort in affected children (114). Additionally, hepatoprotective properties from triterpenoids support liver health, which is crucial for children suffering from conditions like hepatitis or those experiencing drug-induced liver damage (115, 116).

*Allium sativum*, commonly known as garlic, possesses a variety of sulphur compounds, particularly allicin (82). The allicin compound has been shown to promote vasodilation, which can help improve blood flow and lower blood pressure, contributing to better cardiovascular health in children (117, 118). Additionally, allicin is reported to play a role in reducing cholesterol levels, thereby supporting healthy artery function and reducing the risk of heart-related issues (119). Therefore, for children with conditions such as obesity or metabolic syndrome, the incorporation of garlic and its allicin content can aid in managing risk factors associated with cardiovascular diseases, fostering a foundation for healthier growth and development (120, 121).

Furthermore, *Fallopia convolvulus*, *Prunus persica*, *Vachellia karroo*, and *Scabiosa columbaria* are noted for their phenolic compounds (93, 122, 123). The presence of these phenolic compounds in medicinal plants has been associated with substantial antioxidant properties, which are essential in the management and treatment of childhood diseases. These compounds help to neutralize free radicals, which are unstable molecules that can cause oxidative stress and damage cells, potentially leading to various health issues (124, 125). For children, who are particularly vulnerable to oxidative damage, the antioxidant properties of phenolic compounds can support the immune system, reduce inflammation, and enhance overall health (126). By mitigating oxidative stress, these compounds can play a role in preventing and managing conditions such as respiratory diseases, allergies, and even certain metabolic diseases, thereby contributing to improved health outcomes in pediatric populations.

*Senna occidentalis* is recognized for its terpenes like cyperene and *β*-caryophyllene (100). These compounds possess anti-inflammatory, antibacterial, and antiviral properties, which can be beneficial in managing conditions such as respiratory infections, skin diseases, and gastrointestinal diseases (127). For instance, the anti-inflammatory effects of cyperene may help alleviate symptoms of asthma or allergic reactions, while its antibacterial properties can support the treatment of infections (127–129). Additionally, cyperene's ability to enhance immune response can be advantageous in safeguarding children's health against common infectious diseases (130).

The current literature search revealed that the essential oils derived from *Siphonochilus aethiopicus* contains phytochemical compounds like siphonochilone, 1,8-cineole, cis-allo-ocimene, and (E)-β-ocimene (102). These are terpene compounds and have demonstrated therapeutic potential for several childhood diseases. For instance, 1,8-cineole, known for its expectorant and anti-inflammatory properties, can help in treating respiratory conditions such as asthma and bronchitis (131, 132). Meanwhile, siphonochilone has shown antimicrobial activity, which can be useful for addressing infections common in children (133). Additionally, both cis-allo-ocimene and (E)-β-ocimene exhibit anti-inflammatory and antioxidant effects that may aid in the management of skin conditions like eczema or dermatitis, enhancing health in pediatric patients (134).

### 3.9 Antibacterial activity of the documented medicinal plants

The current literature review has identified 31 out of the 38 medicinal plants utilized in the Ehlanzeni District for the treatment of childhood diseases that have been scientifically validated for their antibacterial properties (Table 3). Among these, five plants demonstrated particularly high antibacterial activity, with Minimum Inhibitory Concentration (MIC) values below 15 µg/mL. According to Kowalska-Krochmal and Dudek-Wicher (162); Rodríguez-Melcón et al. (163), lower MIC values are preferred, as they indicate that smaller quantities of the antimicrobial agent are needed to inhibit microbial growth.

**Table 3:** Antibacterial activities of medicinal plants used in the Ehlanzeni District, Mpumalanga Province, South Africa.

S/N	Name of plant	Bacterial strain and MIC
1.	*Abrus precatorius*	*Staphylococcus aureus*: 1,560 to 3,130 µg/mL (135)
2.	*Acokanthera oppositifolia*	*Pseudomonas aeruginosa*: 7.81 μg/mL
		*Escherichia coli*: 0.98 μg/mL (76)
3.	*Acorus calamus*	*E. coli*: 16,000 to 42,000 μg/mL (77)
4.	*Alepidea amatymbica*	390 µg/mL against *Bacillus subtilis* (136)
5.	*Allium sativum*	*E. coli*: 625 µg/mL
		*S. aureus*: 12,500 µg/mL (137, 138)
6.	*Aloe ferox*	100 to 500 µg/mL against *Neisseria gonorrhoeae* (83)
7.	*Ansellia africana*	*Mycobacterium smegmatis* and *S. aureus*, 2,500 to 10,000 µg/mL (139)
8.	*Artemisia afra*	2,600 to 9,300 µg/mL against *Enterococcus faecalis, Moraxella catarrhalis, Klebsiella pneumonia* (140)
9.	*Bulbine frutescens*	125 µg/mL against *Cutibacterium acnes* (141)
10.	*Carica papaya*	*P. aeruginosa*, 12.5 mg/mL (142)
11.	*Clematis brachiata*	*S. aureus, Staphylococcus epidermidus, Bacillus cereus, Microbacterium kristinae,* and *S. faecalis* 1,000 to 3,000 µg/mL (89)
12.	*Coix lacryma-jobi*	0.031 g/L against E. coli, S. aureus, and B. subtilis (90)
13.	*Cussonia transvaalensis*	0.02 µg/mL against *S. epidermidis* and *S. aureus* (91)
14.	*Diospyros mespiliformis*	*Propionibacterium acnes*: 50 µg/mL (143)
		*Trichophyton mentagrophytes*: 100 µg/mL (143)
15.	*Euclea crispa*	*P. aeruginosa*: 310 and 1,250 µg/mL (144)
16.	*Gomphocarpus fruticosus*	*S. aureus*: 1,000 µg/mL (145)
		*E. coli*: 6,000 µg/mL (145)
17.	*Gymnosporia senegalensis*	*E. coli*: 78 to 1,250 μg/mL (146)
18.	*Hypoxis hemerocallidea*	*S. aureus, E. faecalis, E. coli, P. aeruginosa*: 630 µg/mL (147)
19.	*Lannea schweinfurthii*	*B. cereus*: 500 μg/mL, (148)
20.	*Lantana rugosa*	*E. coli, P aeruginosa, E faecalis* and *S. aureus*: 400 to 1,600 µg/mL (149)
21.	*Leonotis ocymifolia*	*Klebsiella pneumonia*: 130 µg/mL (97)
22.	*Lippia javanica*	40 µg/mL against *P. aeruginosa* and 280 to 350 µg/mL against *S. aureus*: (150)
		4,000 µg/mL against *K. pneumoniae* (151)
23.	*Lycopersicon esculentum*	31,250 µg/mL against *S. aureus,* and 62,500 µg/mL *against K. pneumoniae* and *Shigella flexneri* (152)
24.	*Prunus persica*	62.50 µg/mL against *S. aureus* (153)
25.	*Scabiosa columbaria*	No notable activity (154)
26.	*Schizocarphus nervosus*	*S. aureus*: 40,000 µg/mL
		*K. pneumoniae*: 60,000 µg/mL (155)
27.	*Sclerocarya birrea*	*S. aureus, P. aeruginosa, E. coli* and *E. faecalis*: 150 to 3,000 µg/mL (156)
28.	*Senna occidentalis*	*E. coli* and *P. aeruginosa*: 12,500 µg/mL
		*S. aureus*: 6,250 µg/mL (157)
29.	*Sida acuta*	3,130 µg/mL against *S. aureus* (158)
30.	*Vachellia karroo*	*S. aureus*, *E. faecalis*, *E. coli*, *P. aeruginosa, K. pneumoniae*, 7.5 to 250 μg/mL (159)
		*E. coli, K. pneumoniae, P. aeruginosa* and *S. aureus*, 78.12 to 1,250.00 μg/mL (160)
31.	*Warburgia salutaris*	*S. aureus* and *B. subtilis* 12.5 µg/mL (161)

The plant with the highest activity (low MIC value) was *Cussonia transvaalensis* with an MIC value of 0.02 µg/mL against *Staphylococcus epidermidis* and *Staphylococcus aureus* (91). The reported efficacy of *C. transvaalensis* against these two bacterial strains indicates its potential value in the treatment of several childhood conditions, particularly those associated with skin infections. Conditions such as boils, which are often caused by *S. aureus*, and impetigo, a highly contagious skin infection commonly seen in young children, may be effectively addressed using this plant (164, 165). Additionally, it could help manage folliculitis, an inflammation of the hair follicles, as well as other skin irritations that arise from *S. epidermidis* and *S. aureus*. He and Wunderink (166) further noted that *S. aureus*, particularly in its methicillin-resistant form (MRSA), can cause pneumonia, and sometimes serious bloodstream infections. In rural areas, limited access to healthcare facilities, lack of medical resources, and insufficient vaccination coverage can exacerbate the risks associated with these infections (167, 168). Children in these regions are often more vulnerable due to factors such as malnutrition and higher exposure to environmental pathogens (169). Severn and Horswill (170) indicated that *S. epidermidis* presents significant health concerns, primarily due to its ability to form biofilms that enhance its resistance to treatment options. This characteristic heightens the potential value of utilizing natural plant-based treatments in the management of this pathogen and related infections.

*Coix lacryma-jobi* showed great antibacterial potency with an MIC value of 0.031 g/L against *Escherichia coli (E. coli)* and *Bacillus subtilis (B. subtilis)* (90). Similarly, the study by Rabe and Van Staden (161) showed that *Warburgia salutaris* is active against *B. subtilis* with an MIC value of 12.5 µg/mL. *E. coli* is often associated with gastrointestinal infections, leading to diarrhea, abdominal pain, and in severe cases, dehydration or hemolytic uremic syndrome (171). *B. subtilis* is less common in causing direct infections but can be associated with foodborne diseases in children, particularly through contaminated food products (172). By effectively targeting *E. coli* and *B. subtilis*, *Coix lacryma-jobi* and *W. salutaris* may help inhibit the growth of these pathogens and could play a crucial role in managing infections and improving child health outcomes.

The research conducted by El Sayed et al. (76) and Dagne et al. (142) have demonstrated that *Acokanthera oppositifolia* and *Carica papaya* exhibits substantial antimicrobial activity *against Pseudomonas aeruginosa (P. aeruginosa)*, with MIC values of 7.81 μg/mL and 12.5 mg/mL, respectively. *P. aeruginosa* is commonly associated with respiratory infections, particularly in pediatric patients with underlying conditions such as cystic fibrosis or bronchopulmonary dysplasia; it can also lead to skin infections such as rashes or lesions and urinary tract infections (173, 174). These infections represent significant threats to the health of children, underscoring the need for effective treatment options. Given the potent activity of extracts from *A. oppositifolia* and *C. papaya* against these pathogens, they may serve as viable antimicrobial agents, offering a natural and potentially cost-effective alternative for treating infections caused by *P. aeruginosa*. Therefore, the use of these medicinal plants may improve health outcomes for affected children, particularly in rural areas where access to modern healthcare services is limited (50). The incorporation of such indigenous botanical agents emphasizes the crucial role of traditional medicine practices in effective disease management within pediatric populations.

### 3.10 Antifungal activity of medicinal plants used for paediatric care

This study revealed that out of the 38 medicinal plants utilized in the Ehlanzeni District of the Mpumalanga Province for addressing childhood diseases, 25 have been previously assessed for their antifungal properties (Table 4). Most of these plants demonstrated antifungal efficacy, with four exhibiting MIC values of 20 µg/mL or lower. A total of 16 medicinal plants were specifically evaluated against *Candida albicans (C. albicans)*, yielding MIC values that varied from 3.9 to 6,900 µg/mL. *Acokanthera oppositifolia* emerged as the most potent antifungal agent, displaying an MIC value of 3.9 µg/mL against *C. albicans* (76).

**Table 4:** Antifungal activities of medicinal plants used in the Ehlanzeni District, Mpumalanga Province, South Africa.

S/N	Name of plant	Fungal strain and MIC
1.	*Abrus precatorius*	*Fusarium solani* and *Alternaria solani*: 10.36 µg/mL (175)
2.	*Acokanthera oppositifolia*	*Candida albicans*: 3.9 μg/mL (76)
3.	*Acorus calamus*	*C. albicans*: 500 µg/mL (79)
4.	*Agapanthus africanus*	*Trichophyton mentagrophytes* and *Sporothrix schenekii:* 15.6 µg/mL (80)
5.	*Alepidea amatymbica*	*C. albicans*: 880 µg/mL (136)
6.	*Allium sativum*	40 to 80 μg/mL *against C. albicans* and *Candida tropicalis* (176)
		2,500 µg/mL against *Fusarium spp.* (177)
		1,500 µg/mL for *Aspergillus flavus* and 3,000 µg/mL for *Aspergillus niger* (178)
7.	*Aloe ferox*	*C. albicans*: 5,000 µg/mL (179)
8.	*Artemisia afra*	20 to 625 µg/mL against *C. albicans* (180)
9.	*Bulbine frutescens*	*C. albicans*: 6,900 (181)
10.	*Carica papaya*	*C. albicans*, 350 μg/mL (182)
11.	*Clematis brachiata*	*A. niger, A. flavus,* and *Penicillium*
		*Notatum*: 10,000 µg/mL (89)
12.	*Gymnosporia senegalensis*	*Cryptococcus neoformans*: 39 to 1,250 μg/mL (146)
13.	*Hypoxis hemerocallidea*	*Trichophyton tonsurans*: 780 µg/mL (95)
14.	*Lantana rugosa*	*C. albicans* and *C. neoformans*: 2,000 µg/mL (183)
15.	*Leonotis ocymifolia*	*Candida glabrata*: 130 µg/mL (97)
16.	*Lippia javanica*	*C. albicans*: 3,280 µg/mL (184)
17.	*Lycopersicon esculentum*	*A. niger*: 600 µg/mL (185)
		*C. albicans*, 500 μg/mL (186)
18.	*Prunus persica*	62.50 µg/mL against C*. albicans*, (153)
19.	*Schizocarphus nervosus*	*C. albicans*: 4,000 µg/mL (155)
20.	*Sclerocarya birrea*	*Candida albidus*: 170 µg/mL (187)
21.	*Senna occidentalis*	600 μg/mL against *C. albicans* (188)
		200–1,000 μg/mL against *C. albicans* (189)
		78–312 μg/mL against *A. niger* (100)
22.	*Sida acuta*	*C. albicans*: 78 to 625 µg/mL (101)
23.	*Siphonochilus aethiopicus*	*Fusarium oxysporum*: 78 to 312,5 µg/mL (190)
24.	*Vachellia karroo*	*C. albicans:* 800 µg/mL (191)
		*C. albicans and M. audouinii:* 78.12 μg/mL (160)
		*C. albicans:* 1,600 µg/mL (192)
25.	*Warburgia salutaris*	*Aspergillus parasiticus, Aspergillus ochraceus, A. flavus, Fusarium graminearum, F. oxysporum, Fusarium verticillioides*: 130 µg/mL (193)

*C. albicans* is a fungal organism associated with several pediatric conditions, especially oral thrush and diaper rash (194). These conditions can cause significant discomfort for young children. Oral thrush is characterized by the presence of white patches in the oral cavity, which may result in pain and difficulties during feeding, while diaper rash is identified by red, inflamed skin in the diaper area. Recent studies have highlighted a growing interest in the antifungal properties of medicinal plants, such as *A. oppositifolia* (195, 196). This plant is recognized for its strong antifungal and antimicrobial effects and has been utilized in traditional medicine across various cultures to treat infections (197, 198). Research findings by El Sayed et al. (76) suggest that extracts derived from *A. oppositifolia* exhibit the ability to inhibit the growth of Candida species, thereby presenting a promising natural alternative treatment for managing childhood conditions associated with *C. albicans*.

Yasmin et al. (175) conducted a study evaluating the antifungal properties of *Abrus precatorius* against the pathogens *Fusarium solani* and *Alternaria solani.* The findings revealed that *A. precatorius* exhibited antifungal activity, with MIC value of 10.36 µg/mL. *F. solani* and *A. solani* are fungal pathogens known to cause various diseases in children, particularly in immunocompromised individuals (199). *F. solani* is often associated with skin infections, such as superficial mycoses, which can lead to discomfort and secondary infections if untreated (200). On the other hand, *A. solani* can contribute to allergic reactions and respiratory issues, particularly in young children who may be sensitive to environmental allergens (201). For these fungal infections, natural potent antifungal agents, such as extracts derived from *A. precatorius* may be effective. This plant is recognized in traditional medicine for its antifungal properties (202–204). Furthermore, its low MIC value, as indicated by Yasmin et al. (175), indicates that it may effectively inhibit the growth of these fungi, making it potentially valuable for further research and applications in antifungal treatments.

*Agapanthus africanus* showed antifungal activity against *Trichophyton mentagrophytes* and *Sporothrix schenekii* with an MIC value of 15.6 µg/mL (80). *T. mentagrophytes* is primarily responsible for tinea infections, such as athlete's foot and ringworm, which can manifest as itchy, red, and scaly patches on the skin or scalp of affected children (205, 206). *S. schenekii* primarily causes sporotrichosis in children, which can manifest as skin lesions, lymphocutaneous infections, and, in severe cases, disseminated infections affecting internal organs (207). The treatment of these conditions often involves the use of potent antifungal agents, one of which is *A. africanus*. Research has indicated that extracts from this plant possess antifungal properties, making it a valuable option for combating infections caused by these pathogens, particularly in pediatric populations where effective treatment is crucial.

*Allium sativum* has demonstrated moderate antifungal activity against *Candida tropicalis*, with MIC ranging from 40 to 80 μg/mL, as reported by Uzun et al. (176). *C. tropicalis* is a pathogenic yeast associated with various diseases, particularly in children. Conditions related to *C. tropicalis* include candidiasis, which may present as oral thrush, esophageal candidiasis, or invasive candidiasis impacting the bloodstream (208, 209). Vila et al. (210) cited that young children, especially those with pre-existing health issues, face a significantly increased risk of these infections. Furthermore, *A. sativum* is extensively utilized in traditional medicine for treating various infections caused by candida species, as indicated by Abirami et al. (211); Khounganian et al. (212); Sasi et al. (213). These findings suggest that *A. sativum* may serve as a potential treatment option for infections caused by Candida tropicalis.

*Vachellia Karroo* showed antifungal activity against *Microsporum audouinii* with an MIC value of 78.12 μg/mL (192). *M. audouinii*, a fungus predominantly associated with skin infections, is primarily known for causing tinea capitis, commonly referred to as scalp ringworm, which affects children (214). This condition can lead to symptoms like itchy, scaly patches on the scalp and, in more severe cases, hair loss. Although often self-limiting, tinea capitis can lead to secondary bacterial infections if not addressed (215). Potent antifungal agents, including natural products like *Vachellia karroo*, have shown promising antifungal activity against pathogens like *M. audouinii*, with a low MIC value indicating its effectiveness (192). The use of *V. karroo* offers a potential alternative treatment option for this disease, especially in cases where conventional antifungals may not be suitable due to resistance or side effects. Utilizing such natural treatments may be especially advantageous in the management of this childhood disease. This approach not only offers a holistic methodology to treatment but also has the potential to lower costs and decrease dependence on synthetic antifungal agents.

### 3.11 Cytotoxicity and safety profiles of the identified plant species

This study revealed that among the 38 medicinal plants utilized for the treatment of childhood diseases in the Ehlanzeni District of Mpumalanga Province, 25 have undergone scientific validation for their cytotoxic effects against various cell lines (Table 5). Cytotoxicity analysis of medicinal plants is essential in evaluating their safety and efficacy for treating childhood diseases, as it helps to identify potential toxic effects on healthy cells while determining therapeutic benefits (232). This analysis ensures that herbal treatments not only offer effective treatment options but also minimize risks to vulnerable pediatric populations (233). This study discovered that 36% of the plants used in the Ehlanzeni District demonstrated cytotoxic effects, with IC50 values below 15 μg/mL. One of these plants, *Agapanthus africanus* exhibited a significant cytotoxic effect against SBC-3 cells (Human Small-Cell Lung Cancer), achieving an IC50 value of 3.7 ± 0.033 μg/mL as indicated by Iguchi et al. (218). This finding indicates that *A. africanus*, a medicinal plant commonly utilized in traditional medicine, presents significant potential as a therapeutic option for treating cancers with neuroendocrine features similar to the SBC-3 line.

**Table 5:** Reported cytotoxicity of medicinal plants used to treat childhood diseases in the Ehlanzeni District, Mpumalanga Province, South Africa.

S/N	Plant name	Cell line and IC_50_ value
1.	*Acokanthera oppositifolia*	HepG2 cells: 24.26 µg/mL and 26.16 µg/mL
		MCF-7 cells: 12.50 µg/mL and 15.00 µg/mL (197, 216)
2.	*Acorus calamus*	MDA-MB-435S: 63.65 ± 8.30 μg/mL
		Hep3B cell lines: 85.22 ± 11.40 μg/mL (78)
3.	*Adenia gummifera*	KB human cell lines: 1.1 µg/mL (217)
4.	*Agapanthus africanus*	SBC-3 Cells (Human Small-Cell Lung Cancer): 3.7 ± 0.033 μg/mL (218)
5.	*Allium sativum*	2.11 µg/mL against Caco-2 cells (colon cancer) (219)
		21.47 µg/mL and 4.49 µg/mL against MCF-7 cells (breast cancer) (219)
		45.12 µg/mL and 0.95 µg/mL against HepG-2 cells (liver cancer) (219)
6.	*Ansellia africana*	Vero cells: 25.64 µg/mL (85)
7.	*Artemisia afra*	178.47 µg/mL against Vero cells (86)
8.	*Carica papaya*	HepG2 liver cancer cells: 20 μg/mL (220)
9.	*Coix lacryma-jobi*	HeLa: 23.8 μg/mL
		HepG2: 67.6 μg/mL and SGC-7,901 cells: 25.5 μg/mL (221)
10.	*Diospyros mespiliformis*	73.27, 110.55 and 109.8 µg/ mL, respectively, for HUH7, Hep3B and HEPA-RG cells (222)
11.	*Fallopia convolvulus*	122.9 ± 6.98 µg/mL against HeLa cervical cancer cells (94)
12.	*Gomphocarpus fruticosus*	HepG2: 0.07 µg/mL (223)
13.	*Gymnosporia senegalensis*	Vero cell lines: 87 to 187 μg/mL (146)
14.	*Lantana rugosa*	C3A liver cell lines: .4 μg/mL to 6.4 μg/mL (150)
15.	*Leonotis ocymifolia*	175 µg/mL against Jurkat leukemia cells and 133 µg/mL against HL60 leukemia cells (224)
16.	*Lippia javanica*	C3A liver cells: 0,01 to 359,4 μg/mL (150)
17.	*Lycopersicon esculentum*	Caco-2 cells: 9.69 ± 0.6 µg/mL
		MCF-7 cells: 12.52 ± 0.58 µg/mL
		HepG2 cells: 14.34 ± 0.62 µg/mL (225)
18.	*Prunus persica*	HeLa cells: 12.22 µg/mL
		MPanc-96 cells: 28.17 µg/mL
		MCF-7 cells: 35.51 µg/mL (153)
19.	*Scabiosa columbaria*	no significant cytotoxicity against human dermal fibroblasts MRHF cells (226)
20.	*Schizocarphus nervosus*	HepG2 cells: 30 ± 5 μg/mL (227)
21.	*Sclerocarya birrea*	HepG2: 180 to 270 µg/mL (228)
22.	*Senna occidentalis*	7.88 µg/mL against AChE
		3.78 µg/mL against BChE (229)
23.	*Sida acuta*	HeLa cells (human cervical cancer): 610.00 ± 2.5 µg/mL (230)
24.	*Vachellia karroo*	Vero African monkey cells: 115 μg/mL (192)
25.	*Warburgia salutaris*	MCF-7 cell line, 34.15 *μ*g/mL (231)

Previous studies have demonstrated the ability of *A. africanus* to induce apoptosis in cancer cells and inhibit tumor growth (218, 234, 235). SBC-3 cells are derived from human small-cell lung cancer and can present characteristics that overlap with several childhood diseases, particularly certain hematological malignancies and neuroblastomas (236). While small-cell lung cancer predominantly affects adults, research has indicated that cancers in children may similarly express neuroendocrine features, making the study of SBC-3 cells relevant (237, 238).

The study by Cordier et al. (197) and Fouché et al. (216) found that *Acokanthera oppositifolia* showed cytotoxic effects against MCF-7 cells, with IC50 values of 12.50 µg/mL and 15.00 µg/mL, respectively. MCF-7 cells are a widely used human breast cancer cell line that originates from a metastatic breast tumor in a 69-year-old woman (239). While MCF-7 cells themselves are not directly linked to childhood diseases, their study has implications for understanding certain pediatric conditions. Research involving MCF-7 cells can shed light on the mechanisms of breast cancer, including interactions with hormones and potential genetic factors that may influence cancer development in individuals, including children. Although breast cancer is rare in children, understanding the biology of breast cancer using MCF-7 cells can lead to insights into rare childhood tumors, such as phyllodes tumors or other sarcomas that may arise in breast tissue (240, 241). Moreover, studies on MCF-7 cells can inform on therapeutic approaches that could be beneficial in developing treatments for pediatric patients with similar oncogenic pathways. Thus, while MCF-7 cells are primarily used for adult cancer research, they play a role in the broader understanding of cancer biology that can indirectly impact childhood disease research.

*Adenia gummifera* demonstrated a cytotoxic effect against KB human cell lines, with a reported IC50 value of 1.1 µg/mL, as indicated in the study by Fullas et al. (217). Both Fullas et al. (217) and Mouafon and Katerere (242) observed that extracts from *A. gummifera* can induce apoptosis in cancer cells and inhibit tumor growth. These findings suggest the potential for these extracts to enhance the effectiveness of conventional chemotherapy and reinforce *A. gummifera's* efficacy against conditions related to KB human cell lines. KB human cell lines are derived from a human oral squamous cell carcinoma and serve as valuable models in cancer research. They are particularly instrumental in advancing our understanding of various childhood diseases, especially those associated with head and neck cancers (243, 244). In pediatric oncology, conditions such as rhabdomyosarcoma and other soft tissue sarcomas often exhibit similarities to the characteristics of KB cells, especially regarding their aggressive behavior and metastatic potential (245). The insights gained from KB cells help researchers explore therapeutic interventions and the mechanisms of drug resistance to improve treatment strategies for childhood cancers and related diseases. Furthermore, the study of KB cell responses to various chemotherapeutic agents could provide crucial information for developing targeted treatments that may lessen toxicity and enhance efficacy in managing pediatric cancers.

Ahmed et al. (219) evaluated *Allium sativum* for its cytotoxic effect against Caco-2 cells (colon cancer) and discovered that the plant showed potency with a reported IC50 value of 2.11 µg/mL against the cells. Caco-2 cells, derived from human colon adenocarcinoma, are widely used in cancer research and have implications for understanding various childhood diseases, particularly gastrointestinal malignancies such as colon cancer (246). Though colon cancer is rare in young children, conditions like familial adenomatous polyposis (FAP) and colorectal carcinomas can present in pediatric populations, especially in hereditary contexts (247). Caco-2 cells serve as models to study the molecular mechanisms underlying these diseases and to evaluate therapeutic interventions. By integrating the use of naturally effective cytotoxic agents such as *A. sativum*, there is a possibility for developing more effective treatment strategies for childhood gastrointestinal cancers. This approach not only targets tumor cells directly but may also enhance overall patient outcomes by reducing treatment-related side effects typically associated with standard chemotherapy.

Recent scientific validation has confirmed the cytotoxic effects of seven medicinal plants used for treating childhood diseases in the Ehlanzeni District, specifically against HepG2 cell lines. Among these, *Gomphocarpus fruticosus* emerges as a particularly promising candidate for further exploration. A study conducted by Mokbel et al. (223) assessed the cytotoxic impact of extracts from *G. fruticosus* on HepG2 cell lines, revealing a substantial cytotoxic effect with an IC50 value of 0.07 µg/mL. HepG2 cell lines are derived from human liver carcinoma and are valuable for studying various diseases related to liver function and dysfunction, including childhood conditions such as hepatitis and liver tumors (248, 249). Though primary liver cancer is relatively rare in children, it can occur due to factors like congenital biliary atresia or metabolic diseases that affect liver health (250–252). The HepG2 cell line provides researchers with a model to investigate the mechanisms behind these conditions and evaluate potential therapeutic strategies.

The research conducted by Makhafola et al. (150) examined the cytotoxic effects of *Lantana rugosa* and *Lippia javanica*. The findings indicated that both plants exhibit cytotoxic activity against C3A liver cell lines. *L. rugosa* demonstrated IC50 values ranging from 4 μg/mL to 6.4 μg/mL, while *L. javanica* presented IC50 values between 0.01 and 359.4 μg/mL. The investigation of these plants lays the groundwork for further studies into their potential effectiveness in addressing liver-related conditions in pediatric populations. Additionally, these results suggest that extracts from *L. rugosa* and *L. javanica* could play a role in enhancing treatment strategies for childhood diseases linked to liver cell dysfunction by addressing abnormal cell proliferation and promoting improved liver health. C3A liver cell lines play a momentous role in the study of various childhood diseases, particularly those related to liver dysfunction and metabolic diseases (253, 254). One notable condition is viral hepatitis, which can result in liver inflammation and long-term damage in paediatric populations (255). Additionally, certain inherited metabolic diseases, such as tyrosinemia, can also be investigated using C3A cells due to their hepatic origin (256).

## 4 Conclusion

The current study has provided a comprehensive literature search and inventory of medicinal plants used to treat childhood diseases in the Ehlanzeni District, highlighting the phytoconstituents, antibacterial and antifungal, and cytotoxicity properties of the plants. To date, only three studies have investigated the use of medicinal plants for the treatment of childhood diseases in two specific local municipalities within the Ehlanzeni District: Bushbuckridge and Nkomazi. These studies have provided valuable insights into the traditional practices and herbal remedies utilized by local communities in these areas to address various childhood diseases. However, it is noteworthy that the remaining two municipalities within the Ehlanzeni District (Mbombela and Thaba Chweu) have not yet been explored for their medicinal plants used for treating childhood diseases. This gap in research presents an opportunity to uncover potentially valuable knowledge about local indigenous herbal medicine practices in those unexplored communities. Recognizing the potential of these plants is essential to advance toward more effective strategies to enhance health and well-being of children. Additionally, it is vital to document medicinal plants and their associated knowledge for the preservation of invaluable indigenous knowledge and to empower future generations to benefit from these practices. This study sets the stage for further scientific exploration of these plants’ therapeutic applications.
